# Influence of Humic Acids on the Removal of Arsenic and Antimony by Potassium Ferrate

**DOI:** 10.3390/ijerph20054317

**Published:** 2023-02-28

**Authors:** Ning Wang, Wenwen Li, Nannan Wang, Man Li, Hongbo Wang

**Affiliations:** 1 School of Municipal and Environmental Engineering, Shandong Jianzhu University, Jinan 250101, China; 2 Resources and Environment Innovation Institute, Shandong Jianzhu University, Jinan 250101, China; 3 Qingdao Municipal Engineering Design and Research Institute, Qingdao 266061, China; 4 Shandong Soil Pollution Prevention and Recalcination Center, Jinan 250033, China

**Keywords:** potassium ferrate, As(III), Sb(III), mass ratio, humic acid (HA)

## Abstract

Although the removal ability of potassium ferrate (K_2_FeO_4_) on aqueous heavy metals has been confirmed by many researchers, little information focuses on the difference between the individual and simultaneous treatment of elements from the same family of the periodic table. In this project, two heavy metals, arsenic (As) and antimony (Sb) were chosen as the target pollutants to investigate the removal ability of K_2_FeO_4_ and the influence of humic acid (HA) in simulated water and spiked lake water samples. The results showed that the removal efficiencies of both pollutants gradually increased along the Fe/As or Sb mass ratios. The maximum removal rate of As(III) reached 99.5% at a pH of 5.6 and a Fe/As mass ratio of 4.6 when the initial As(III) concentration was 0.5 mg/L; while the maximum was 99.61% for Sb(III) at a pH of 4.5 and Fe/Sb of 22.6 when the initial Sb(III) concentration was 0.5 mg/L. It was found that HA inhibited the removal of individual As or Sb slightly and the removal efficiency of Sb was significantly higher than that of As with or without the addition of K_2_FeO_4_. For the co-existence system of As and Sb, the removal of As was improved sharply after the addition of K_2_FeO_4_, higher than Sb; while the latter was slightly better than that of As without K_2_FeO_4_, probably due to the stronger complexing ability of HA and Sb. X-ray energy dispersive spectroscopy (EDS), X-ray diffractometer (XRD), and X-ray photoelectron spectroscopy (XPS) were used to characterize the precipitated products to reveal the potential removal mechanisms based on the experimental results.

## 1. Introduction

Given that by 2025, two-thirds of the world’s population will experience severe water shortages, securing water usage is one of the crucial concerns for researchers in the 21st century [1]. Heavy metals in the water environment harm aquatic ecosystems [2] and accumulate in food chains causing damage to the liver, the heart, and the nervous system of humans [3]. Arsenic and antimony are two carcinogenic heavy metals that have received significant attention from scientists and researchers [4]. Approximately 188,000 tons of antimony was consumed globally in 2016, about five times as much as arsenic [5]. In recent years, arsenic and antimony pollution incidents have occurred more frequently in major environmental matrices [6,7].

It has been reported that arsenic is naturally present in 31 groundwaters in various nations and areas in Asia, Europe, and the Americas [6]. This groundwater is used by millions of people for drinking and agriculture, thus endangering their health [6]. Artificial arsenic sources include mining, arsenic-containing pesticides, herbicides, and crop desiccants, as well as other processes such as industrial wastewater discharge and chemical waste treatment [8]. Chronic poisoning can occur when a person is exposed to excessive levels of arsenic for an extended period of time, causing the element to accumulate in the skin, hair, and nails [9]. Arsenic primarily exists in the form of arsenate (AsO_4_^3−^) and nitrite (AsO_3_^3−^) in natural water bodies. H_2_AsO_4_^−^ and HAsO_4_^2−^ dominate the aqueous solutions at a pH value of 4–8; while, H_3_AsO_3_ outnumbers other species when the pH is less than 9 under reducing conditions [10]. As(III) is approximately 60 times as toxic as A_S_(V). Previous studies have shown that many thousands of tons of aromatic arsenic compounds are used in veterinary drugs and in feed additives in poultry and swine farms, and released into the environment via solid waste and wastewater discharging [11]. Although the compounds are not highly toxic, they can transform into carcinogenic and highly mobile inorganics containing As(III) and As(V) and threaten the biosafety of the ecosystem [12,13,14].

Sb mostly has two valence states, being Sb(III) and Sb(V), similar to As, in environmental, biological, and geochemical contexts. Antimony exists in the form of Sb(OH)^6−^ under oxidation conditions, and Sb(OH)_3_, Sb(OH)^2+^ and Sb(OH)^4−^ under reducing conditions [15]. Antimony is commonly used in the production of batteries, flame retardants, fabrics, plastics, ammunition, ceramic emulsifiers, and glass decolorants [16], of which approximately 60% is utilized as flame retardants and 20% as alloys [17]. Antimony may cause nausea, vomiting, and diarrhea even with very brief exposure, and it is considered carcinogenic over a longer time frame [18]. Sb(III) is reported to be 10 times more hazardous than Sb(V) [19,20], similar to As(III).

Flocculation is a common technique among various practices for controlling aqueous arsenic and antimony. Guo et al. [21] presented that 98% Sb(V) could be removed by 130 mg/L of ferric chloride flocculant at properly pH value, while As(V) needed only 5 mg/L to achieve the same removal rate. Adsorption is another popular option. Chammui et al. [22] made inexpensive activated carbon from coal mine waste residue to remove As(III) and As(V) from water. However, the adsorption method has restrictions being the low treatment efficiency and treatment of spend adsorbent. Some biological methods, such as sulfate-reducing bacteria (SBR) could reduce Sb(Ⅴ) into Sb (III) and deposit Sb (III) with excessive sulfide in the form of Sb_2_S_3_ [23]. The lengthy microbial culture cycle and toxic inhibition, however, limit the application of this approach. Additionally, since arsenic and antimony are frequently observed co-existing in natural environments [24,25], it is necessary to develop simultaneous elimination techniques. Ferrate has been used in multi-metal ions removal processes. For instance, Liu et al. [26] reported that the combined effect of Fe(VI) and Mn(II) resulted in the adsorption of TI(I) and TI(OH)_3_ by the negatively charged ferric (hydro)oxides/MnO_2_ flocs, achieving the effective removal of trace TI from polluted waters. Song et al. [27] utilized electrolysis to remove both metal elements and found that the highest removal efficiency of arsenic and antimony was 98.69% and 94.45%, respectively, when the electrode distance was 2 cm. Other researchers have attempted to use inexpensive iron-based compounds to extract arsenic and antimony from wastewater. For instance, Sekula et al. [28] constructed an on-site treatment system for arsenic and antimony removal from neutral mine drainage water with zero-valent iron, producing high levels of removal rates (89% for As and 84% for Sb). According to Shan et al. [29], magnetic nanoparticles coated with hematite were effective at removing both As and Sb. This implies that iron-based materials have the potential for the concurrent removal of arsenic and antimony from wastewater. Lan et al. [30] reported a synergistic impact of DP/Ferrate on the simultaneous adsorption of As and Sb.

Natural organic matters (NOMs) exist in most natural waters and most of them are highly sensitive to iron oxides [31]. Additionally, the existence of these dissolved organics would also react with/influence many target pollutants during the treatment by ferrate, such as iodide and total iodine [32], bisphenol A & F, acetaminophen, and 4-tert-butylphenol [33]. Previous studies have demonstrated that NOMs directly impact on the transformation of iron minerals [34]. One significant category of NOMs is humic acid (HA), which influences the removal process of aqueous heavy metals. HA tends to be adsorbed on various mineral colloids, which affects the transport of mineral colloids and the pollutants they adsorbed [35]. It has been reported that a high concentration of HA prevents or delays the transition of Fe(III) in a mineral phase with low crystallization [31,36].

Since the HA concentrations (0–100 mg/L) are many orders of magnitude higher in natural waters than those of heavy metals, this may have a major effect on the formation and surface binding of the ions [37]. Therefore, it is crucial to investigate how HA affects the removal of As and Sb by K_2_FeO_4_. In this project, K_2_FeO_4_ was employed to remove aqueous As(III) and Sb(III), and various influencing factors were investigated, including iron-antimony/-arsenic mass ratio and humic acid (HA). Additionally, the experiments were conducted and compared between in spiked surface water and synthetic water samples.

## 2. Materials and Methods

### 2.1. Reagents and Solutions

100 mg/L arsenic and antimony standard stock solution was prepared in our laboratory and the configuration was as following: (1) 0.1320 g of As_2_O_3_ (analytical grade, Sinopharm Chemical Reagent Co., Ltd., Beijing, China) was dried at 105 °C for 2 h and dissolved in 5 mL of 1 M NaOH (analytical grade, Sinopharm Chemical Reagent Co., Ltd. Beijing, China) in solution. Then 1 M HCl (excellent grade pure, Sinopharm Chemical Reagent Co., Ltd. Beijing, China) was used to neutralize the solution until the red color of phenolphthalein (analytical grade, Tianjin Damao Chemical Reagent Factory, Tianjin, China) faded, and the solution was transferred and diluted in a 1000 mL volumetric flask before being stored in a brown glass bottle at 4 °C; (2) 0.1197 g of Sb_2_O_3_ (analytical grade, Shanghai Yien Chemical Technology Co., Ltd., Shanghai, China) was weighed to prepare stock solution mostly following the previous procedure, except using 80 mL concentrated HCl to dissolve the solid instead of NaOH solution and adding another 120 mL concentrated HCl in the 1000 mL volumetric flask. The K_2_FeO_4_ used in this experiment came from a laboratory of Zhejiang University (purity > 95%), which was synthesized using the method of Thompson et al. [38]. The K_2_FeO_4_ were made by the hypochlorite method using concentrated sodium hypochlorite solution oxidize hydrous ferric oxide, whose precursor was ferric nitrate. Chromite titration [39] was used to check the purity prior of each test. The HA was purchased from Tianjin Guangfu Fine Chemical Research Institute, Tianjin, China (HA Commercial ID: CP500G).

### 2.2. Experiments for Aqueous Heavy Metal Removal

A series of 300 mL simulated water samples (As/Sb dissolved in deionized water) containing certain amounts of As(III)/Sb(III) individually or both were used to carry out the removal experiments by potassium ferrate at room temperature using different dosage mass ratios with HA present or absent. A six-paddle programmable jar tester was set to a quick stir at 250 r/min for 3 min, a slow stir at 60 r/min for 20 min, and then a settlement for 30 min. An amount of supernatant was sampled and filtered using a 0.45 µm microporous filter membrane before being transferred into a colorimetric tube for additional instrument analysis. The remaining As and Sb concentrations in the supernatant was determined using an atomic fluorescence photometer (AFS-933, Beijing Jitian Instrument Co., Ltd., Beijing, China). All glassware used in the process had been soaked in a 10% nitric acid solution for at least 24 h, rinsed with tap water, washed at least three times with deionized water, and dried before usage.

Raw water samples from Yingxue Lake at Shandong Jianzhu University were selected for actual surface water experiments. The characteristics of the water quality were tested and listed in Table S1. The concentrations of As and Sb were 2.55 µg/L and 4.39 µg/L in the raw water, respectively. Therefore, the surface water samples were spiked to adjust both values to 0.5 mg/L in order to prevent increasing comparison test error brought on by low concentrations of the target contaminants.

### 2.3. Surface Characterization

The separated precipitates from the aforementioned experiments were freeze-dried with a freeze dryer (SCIENTZ-10N, Ningbo Xinzhi Biotechnology Co., Ltd., Ningbo, China) for 24 h for surface characterization with X-ray energy spectrometer (EDS, ESCALAB 250Xi, Thermo Fisher Scientific Co., Ltd., Waltham, MA, USA), X-ray diffractometer (XRD, smart lab 3 kw, RIGAKU Co., Ltd., Tokyo, Japan), and X-ray photoelectron spectrometer (XPS, Escalab 250Xi, Thermo Fisher Scientific Ltd., Waltham, MA, USA). EDS was used to characterize the appearance and element content of the solid samples. The precipitate was magnified 10,000 times or 12,000 times. More details can be found in the previous publication [40]. XRD could identify the surface crystalline phase properties. The precipitates were characterized by a diffractometer at 40 kV and 30 mA. The scanning range was 10~90°, with an interval of 0.02°. For XPS, the sample was compressed into 1 cm × 1 cm pellets and a full-spectrum analysis was performed with a monochromatic Alka excitation source (HV = 1486.6 eV, power = 150 W, beam diameter = 400 μm, and C1s were calibrated as 284.8 eV).

## 3. Results and Discussion

### 3.1. The Effect of Mass Ratio on the Removal Effect of Arsenic and Antimony

**Figure 1:**
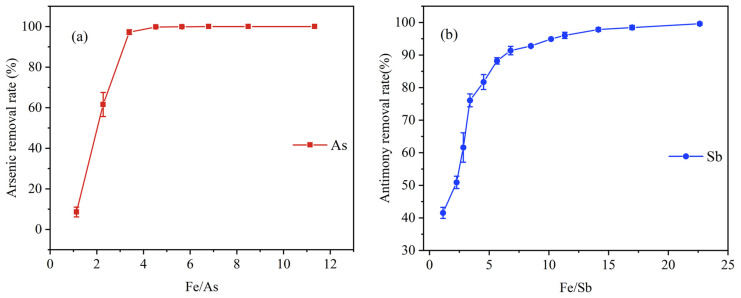
(**a**) Effect of Fe/As mass ratio on removal of individual arsenic, initial As(III) = 0.5 mg/L pH = 6.5 (**b**) Effect of Fe/Sb mass ratio on removal of individual antimony, initial Sb(III) = 0.5 mg/L pH = 4.5.

The effects of Fe/As mass ratios (Fe/As) on As removal rate were investigated, when the initial As(III) concentration was 0.5 mg/L. As can be seen in Figure 1a, the removal efficiency of As increased rapidly as the Fe/As increased until it reached a steady state. The maximum value of 99.55% occurred at Fe/As = 4.6, and the dosage of K_2_FeO_4_ (measured by Fe) of 2.3 mg/L. At this time, the remaining As concentration was less than 0.05 mg/L, which satisfied the requirements of TypeⅠ~III water of the Environmental Quality Standard for Surface Water (GB3838-2002). The removal rate of As increased from 8.58% (Fe/As = 1.1) initially to 97.24% (Fe/As = 3.4), and then gradually increased to 100% (Fe/As = 6.8) with the continuous increase of Fe/As until stabilizing. In the beginning, the ratio of Fe/As was low, resulting in an insufficient amount of K_2_FeO_4_ and with fewer opportunities for contact with As. More K_2_FeO_4_ significantly improved the removal rate of As, as the ratio increased. The results could support the significant effect of K_2_FeO_4_ on the removal of As. It has been reported that the primary mechanism of elimination by K_2_FeO_4_ is the oxidation of As(III) to the less toxic and more easily removable As(V) [41]. The reduced form, Fe(OH)_3_, serves as a potential adsorbent [42]. The reaction equations are presented as follows (Equations (1) and (2)) [40].

(1) $$2{\mathrm{HFeO}}_{4}^{-}+3H_{3}{\mathrm{AsO}}_{3}\to 2{\mathrm{Fe}}^{3+}+H_{3}{\mathrm{AsO}}_{4}^{2-}$$

(2) $${\mathrm{Fe}}^{3+}+3H_{2}O\to \mathrm{Fe}{\left(\mathrm{OH}\right)}_{3}+3H^{+}$$

(3) $${\mathrm{AsO}}_{4}^{3-}+{\mathrm{Fe}}^{3+}={\mathrm{FeAsO}}_{4}↓$$

In addition, Fe(III) also reacted with As(V), which was formed after oxidation, and finally produced iron arsenate (FeAsO_4_) precipitate (Equation (3)). This provides another way for As(III) to be removed. The formed particles showed a large number of lamellar structures, around 30 nm (Figure S1a). It was possibly a dual mechanism resulting in irregular morphology, unlike the spherical particle flocs produced by K_2_FeO_4_ removing Sb (Figure S2a). However, since FeAsO_4_ gradually disappeared under neutral and alkaline conditions [43], As was mostly removed by K_2_FeO_4_ by adsorption.

Figure S1b presents the EDS spectrum of the precipitates formed when K_2_FeO_4_ reacted with aqueous As. It was evident that O, Cl, K, Fe, and As were the most prevalent elements in the deposits. The presence of As confirmed the elimination process carried out by K_2_FeO_4_. In addition, the XRD spectrum demonstrated that the characteristic diffraction peaks at 2θ of 22.15°, 26.71° and 28.77° were the characteristic diffraction peaks of FeAsO_4_ (JCPDS No. 78-1545). This implies that the mechanism of As removed by K_2_FeO_4_ could be attributed to the reduction of Fe(VI) into Fe(III) and the subsequent reaction precipitating FeAsO_4_ between Fe(III) and As(V). Additionally, the characteristic diffraction peak of Fe_3_(AsO_4_)_2_ at 2θ of 40.91° suggested that As was removed by the oxidation of K_2_FeO_4_ to form Fe_3_(AsO_4_)_2_ in Figure S1c [44].

**Figure 2:**
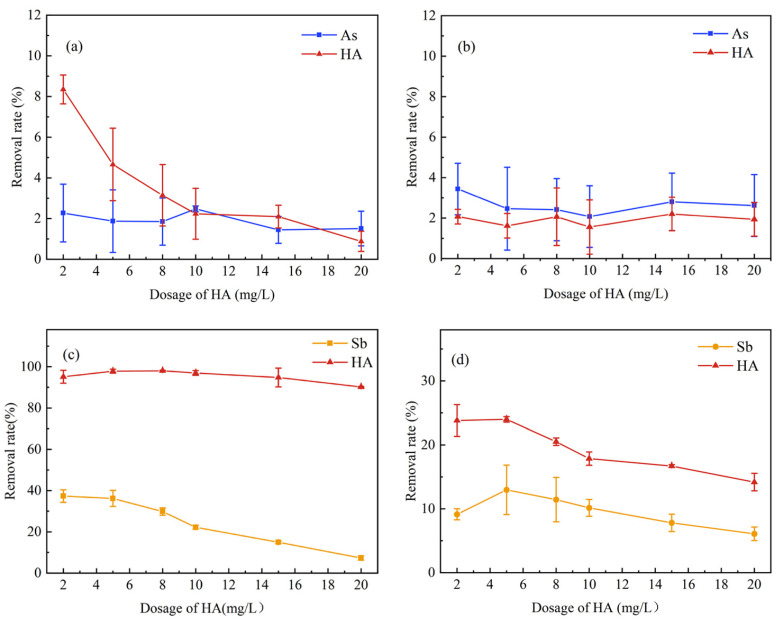
Effect of HA concentration on the removal of As and Sb ((**a**) K_2_FeO_4_(Fe of 2.26 mg/L) +HA + As, pH = 6.5; (**b**) HA + As, pH = 6.5; (**c**) K_2_FeO_4_ (Fe of 3.39 mg/L) + HA + Sb, pH = 4.5; (**d**) HA + Sb, pH = 4.5).

Additionally, Figure 2c shows the characteristic diffraction peaks of Fe(OH)_3_ (2θ of 41.15°) and FeO(OH) (2θ of 40.89°), indicating that the reduction process of K_2_FeO_4_ might generate ferrous-containing colloid particles with adsorption potential. The flocculation caused by K_2_FeO_4_ is attributed to the single charged Fe(OH)_3_ colloid as well as a complex form of iron (hydrogen) oxide compound [45]. Some researchers have already found that the hydrolysis product of K_2_FeO_4_ is a 2-linear ferrihydrite with the smallest crystalline form of iron (hydrogen)oxide [46]. Ferrihydrite has a relatively high surface area and a large number of binding sites for complexing with heavy metals. T Adsorption, specifically the occurrence of a surface complexation event, may be the primary determinant of As removal [30]. The XPS full-scan spectrum (Figure S1d) also showed that Fe, O, K, C, and As were the main elements in the precipitates, consistent with the conclusions from the EDS spectrum (Figure S1b).

As shown in Figure 1b, the profile was similar to As, gradually increasing at the beginning until stabilizing at the initial Sb(III) concentration of 0.5 mg/L. The removal efficiency of Sb was sharply improved to more than 90% as the ratio increased to 6.8. When Fe/Sb was 22.6, namely the dosage of K_2_FeO_4_ (calculated as Fe) of 11.31 mg/L, the removal rate reached 99.61%, while the remaining Sb in the solution was less than 0.005 mg/L, meeting the requirements of the “Surface Water Centralized Drinking Water Sources” (GB 3838-2002) and the “Emission Standards for Tin, Antimony and Mercury Industrial Pollutants” (GB 30770-2014). The pH of 4.5 was chosen with reference to the previous study of Wang et al. [40]. When the pH values were between 4 and 5, K_2_FeO_4_ showed better and more constant removal of Sb with a removal efficiency close to 70%. Since the initial concentration of Sb was constant, more K2FeO4 was dosed as the ratio of Fe/Sb increased. A greater amount of Fe(OH)_3_ was also produced by the reduction of K_2_FeO_4_ in the solution, resulting in a more intense adsorption effect on the Sb ions. In Figure S2c, the diffraction peaks of Sb_2_O_5_ were found at 2θ of 28.77°, 75.66° and 84.38°, suggesting the oxidation of Sb(III) to Sb(V) by K_2_FeO_4_. Clearly, the removal consumed a greater amount of K_2_FeO_4_. Sb(Ⅴ), formed by oxidation by K_2_FeO_4_, was reportedly less ready to be adsorbed than As(Ⅴ) [40]. It may be due to the fact that the ionic radius of Sb is larger than that of As [47], requiring more adsorbent to attach, and the amount of K_2_FeO_4_ added in the process of removing Sb is larger than As. Based on these XPS plots (Figures S1d and S2d) and previous research [40], the binding energies of 45.6 eV and 530.1 eV are assigned to As 3d [48] and Sb 3d [49], respectively, indicating the formation of As (V) and Sb (V) on the surface of the K_2_FeO_4_ hydrolysate. Additionally, the higher content of Cl and Na elements were also found in the EDS plot (Figure S2b), possibly due to the addition of excessive HCl to inhibit the hydrolysis of antimony ions during the preparation of the stock solution, and the addition of NaOH to neutralize it in the later process of the pH adjustment. Meanwhile, the stronger diffraction peaks at 2θ of 32.12°, 45.86° and 56.85° in the XRD pattern (Figure S2c) were the characteristic diffraction peaks of NaCl (JCPDS No. 88-2300), which was consistent with the results of the EDS pattern.

### 3.2. The Effect of Humic Acid on the Removal of Aqueous Antimony and Arsenic

Figure 2a,b showed the impact of HA on K_2_FeO_4_ removing As. It could be seen that compared with zero dosage, the addition of K_2_FeO_4_ had no significant effect on the removal rate of As, which was less than 4% for both circumstances. It could be attributed to the insufficient dosing of K_2_FeO_4_, which was mostly consumed by the HA first. As shown in Figure 2a,b the lower concentrations of HA were more influenced by K_2_FeO_4_. HA contains a series of negatively charged macromolecules, possibly altering the surface charge and the transport retention characteristics of iron mineral colloids [50,51]. As a result, the colloids may develop a negatively-charged surface and consume some adsorption sites, inhibiting As adhering to the ferrite minerals. In addition, the higher anion binding sites in HA may precipitate with cationic iron at an appropriate dose of K_2_FeO_4_ to form an insoluble complex due to the charge neutralization [52], which facilitates the removal of HA. Therefore, the dosage of K_2_FeO_4_ is generally sufficient under the condition of a low concentration of HA, which could be removed to a certain extent in the solution. As the HA concentration increased, the removal rate gradually decreased possibly due to the depletion of K_2_FeO_4_. Overall, the interaction between HA and K_2_FeO_4_ can inhibit the removal of As.

The influence of HA on the removal of Sb could be seen in Figure 2c,d. Similar to the elimination of As, the presence of HA also inhibited the removal process of K_2_FeO_4_ on Sb, since the removal efficiency gradually declined with the HA concentration. This could possibly be attributed to hydrophobic HA molecules competing for the active sites by forming stable complexes on the surface of the precipitates containing iron. However, the removal rates were much higher for Sb and HA than those found in the experiments for As, as can be seen by comparing Figure 2a,c. It is unclear whether the combination between Sb and HA would facilitate the removal of both substances with K_2_FeO_4_. The higher removal rate could be explained by the fact that the core of the Sb(III) molecule has stronger cationic characteristics than As(III), resulting in a better combination with HA through complexation [53] and mutually promoting the removal for both. Therefore, both rates were also higher than As in the control group without any iron mineral (Figure 2d). A further explanation may be that HA has the potential to strongly alter the distribution of Sb (particle, colloid, and dissolved portion) and to change the redox morphology of Sb (Sb(V) relative to Sb(III) abundance), when Sb was initially combined with iron (Fe(III)) oxides such as hydrophyte [54]. In Figure 2d, HA had a certain removal effect on Sb(III) in the absence of K_2_FeO_4_, owing to carboxyl, phenolic and amine groups present in HA complexing with Sb(III) through a ligand exchange reaction of the Sb(III) center under acidic conditions [55].

**Figure 3:**
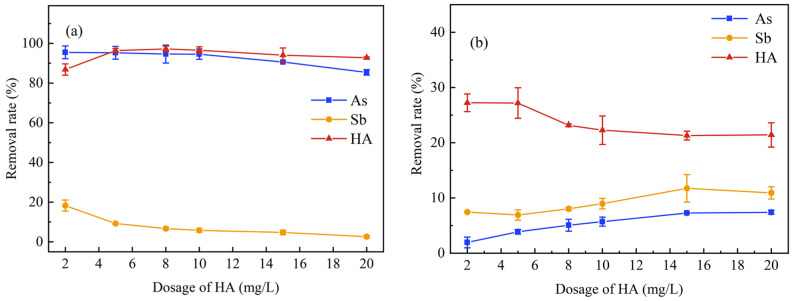
Effect of HA dosing on the simultaneous removal of As and Sb. (**a**) K_2_FeO_4_ (Fe of 8.48 mg/L) treatment; (**b**) no K_2_FeO_4_.

Figure 3a illustrates the effect of the dosage of HA on the simultaneous removal of As and Sb by K_2_FeO_4_. Both HA and As were mostly eliminated while the removal rate of Sb was relatively lower (The highest removal rate was only 18.27%). The removal efficiencies of As and Sb both showed a gradual descent with the increase of HA concentration. Dissolved organic matter may occupy the active sites of the absorbent, thus inhibiting the adsorption of the target pollutants in natural waters [56,57]. Therefore, in-situ reduced ferric particles from K_2_FeO_4_ reduction may be encapsulated by HA, subsequently negatively affecting the removal of As and Sb. In Figure 3b, HA also showed certain removal effects on As and Sb without adding K_2_FeO_4_.

**Figure 4:**
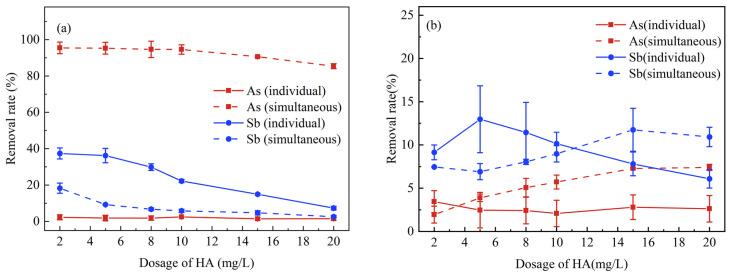
Comparison of HA dosing on the removal of As(III) and Sb(III) between individual and simultaneous treatments ((**a**) K_2_FeO_4_ treatment; (**b**) no K_2_FeO_4_).

Therefore, K_2_FeO_4_ could improve the removal of As and HA as can be seen by comparing Figure 3a,b. According to Figure 4a, it can be seen that the simultaneous removal experiments enhanced the removal of As significantly. Additionally, the dose of K_2_FeO_4_ in the solution was excessive to remove As and Sb simultaneously at low HA concentration, which made it easier to eliminate both As and HA. As was more readily removed than Sb, resulting in a higher efficiency during the simultaneous removal. In Figure 4b, Sb was more easily separated than As during either the individual or simultaneous treatments without K_2_FeO_4_ added. This result may be due to the superior complexing ability of HA and Sb. Previous studies have proposed two mechanisms for HA to complex with Sb(III): (1) Ligand exchange at the center of Sb and release of one or two hydroxides respectively; (2) Formation of negatively charged complexes. In addition, chelation, H-bridge, or cationic metal can stabilize Sb(III) bound to humic acid [55].

### 3.3. The Removal of Arsenic and Antimony by Potassium Ferrate in Surface Water and Simulated Water Samples

**Figure 5:**
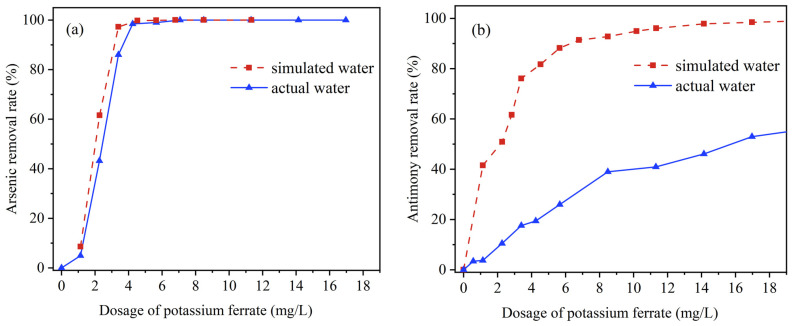
Dosage of K_2_FeO_4_ effect on actual water and simulate water ((**a**) arsenic removal by K_2_FeO_4_, (**b**) antimony removal by K_2_FeO_4_).

The results shown in Figure 5 present the comparison of the removal between the spiked actual surface water and the simulated water. It was found that the removal rates and patterns of As were comparable between the spiked actual water and the simulated water. The removal rate in the simulated water was slightly higher than that in the actual water samples and both curves rose sharply between 1–4 mg/L of K_2_FeO_4_ and. Almost 100% of the heavy metals were removed from both kinds of water samples (Figure 5a) after K_2_FeO_4_ concentration increased to a certain threshold. This indicated that the effect of other pollutants in the actual water body on the removal of As by K_2_FeO_4_ was minimal and essentially negligible. 

However, the removal of Sb was substantially lower in the spiked actual water bodies. The removal rate of Sb in the simulated water increased to 40% when the concentration of K_2_FeO_4_ was increased to 2 mg/L, while 10 mg/L K_2_FeO_4_ was needed for the spiked actual water to reach the equivalent effect (Figure 5b). This phenomenon may be caused by the fact that the presence of other pollutants in the spiked actual water body affected the removal of Sb rather than As. It has been reported that a competitive relationship between Sb and N exists, because of the competition between Sb and N in order to occupy the group-V positions [42]. In addition, Ma et al. [58] found that Sb and atomic-N compete for the same positions under atomic-N-rich conditions. These [42] adversely affect the adsorption efficiency of Sb at the adsorption sites on iron nanoparticles and weaken the removal of Sb.

## 4. Conclusions

The removal of As(III) and Sb(III) by K_2_FeO_4_ was influenced by the Fe/metal mass ratio and HA. The removal rates rose with the ratios and the maximums for As and Sb occurred, respectively, at Fe/As = 4.6 and Fe/Sb = 6.8. The removal rates increased as a function of the ratios. The HA in water could inhibit the removal of As and Sb, mainly because HA formed the colloid with the negatively-charged surface or competed for adsorption sites on the ferrite minerals. In the binary system, the removal performance of As was improved, while Sb was slightly easier to remove if there was no K_2_FeO_4_, possibly due to the strong complexation between HA and Sb. It was found that the removal impact of As was not significantly different in the spiked actual surface water and the simulated water, while it was significantly different for Sb. It is speculated that the substances in the spiked actual water competed for the flocculation of potassium ferrate hydrolysates, which was intended to remove Sb.

## Data Availability

Not applicable.

## Supplementary Materials

The following supporting information can be downloaded at: https://www.mdpi.com/article/10.3390/ijerph20054317/s1, Figure S1: Surface characterization of the precipitation products of individual As removal by 6 K2FeO4 (a: SEM, 10,000 times, b: EDS, c: XRD, d: XPS); Figure S2: Surface characterization of the precipitation products of individual Sb removal by 9 K2FeO4 (a: SEM, 12,000 times, b: EDS, c: XRD, d: XPS); Table S1: Water quality parameters of Yingxue Lake (five samples).
