# Supplementary material for: Influence of Humic Acids on the Removal of Arsenic and Antimony by Potassium Ferrate
Source: Int J Environ Res Public Health. 2023 Feb 28;20(5):4317. doi: 10.3390/ijerph20054317 (PMC10001810; doi:10.3390/ijerph20054317)
Supplement: Supplementary file 1 [file ijerph-20-04317-s001.zip › ijerph-2209610-supplementary.pdf]

International Journal of
Environmental Research and Public Health

MDPI

Influence of Humic Acids on the Removal of Arsenic and Antimony by Potassium Ferrate

Ning Wang, Wenwen Li, Nannan Wang, Man Li and Hongbo Wang

Table S1. Water quality parameters of Yingxue Lake (five samples).

water quality index	numerical value	average value
pH	6.8-6.9	6.9
T/℃	20.3-20.8	20.6
TP/(mg/L)	0.03	0.03
TN/(mg/L)	7.56-7.67	7.60
TOC/(mg/L)	13.6-14.3	14.1
COD/(mg/L)	35.7-36.2	36.0
As/(μg/L)	2.50-2.58	2.55
Sb/(μg/L)	4.35-4.40	4.39

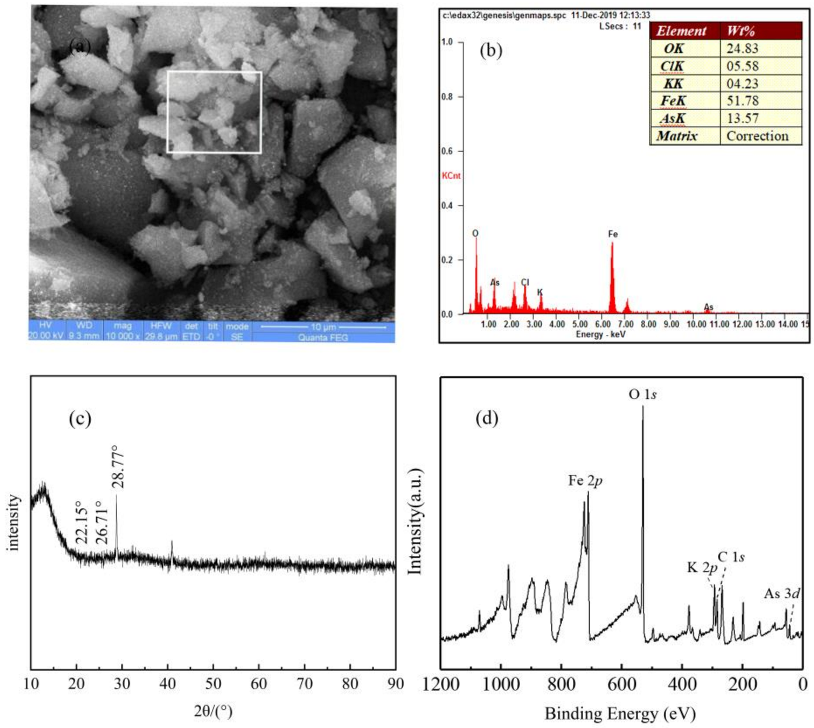

Figure S1. Surface characterization of the precipitation products of individual As removal by K_2FeO_4(a: SEM, 10000 times, b: EDS, c: XRD, d: XPS).

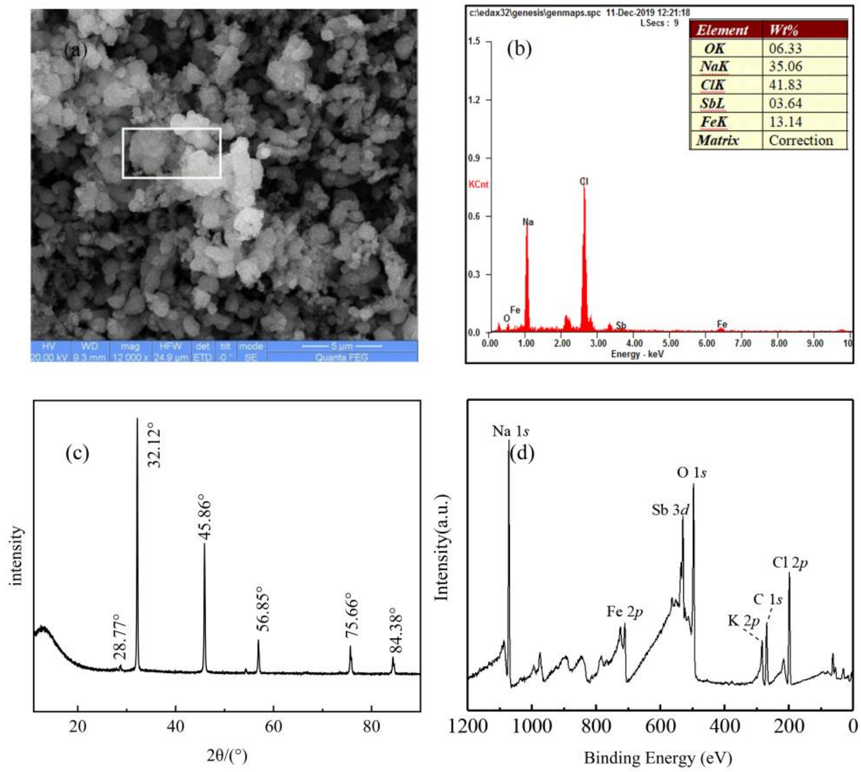

Figure S2. Surface characterization of the precipitation products of individual Sb removal by K2FeO4(a: SEM, 12000 times, b: EDS, c: XRD, d: XPS).
